# VPMBench: a test bench for variant prioritization methods

**DOI:** 10.1186/s12859-021-04458-0

**Published:** 2021-11-08

**Authors:** Andreas Ruscheinski, Anna Lena Reimler, Roland Ewald, Adelinde M. Uhrmacher

**Affiliations:** 1 Modeling and Simulation Group, Institute for Visual and Analytic Computing, University of Rostock, Albert-Einstein-Straße 22, 18051 Rostock, Germany; 2 Limbus Medical Technologies GmbH, Lindenstraße 2, 18055 Rostock, Germany

**Keywords:** Bioinformatics, Software, Test bench, Variant prioritization, Evaluation

## Abstract

**Background:**

Clinical diagnostics of whole-exome and whole-genome sequencing data requires geneticists to consider thousands of genetic variants for each patient. Various variant prioritization methods have been developed over the last years to aid clinicians in identifying variants that are likely disease-causing. Each time a new method is developed, its effectiveness must be evaluated and compared to other approaches based on the most recently available evaluation data. Doing so in an unbiased, systematic, and replicable manner requires significant effort.

**Results:**

The open-source test bench “VPMBench” automates the evaluation of variant prioritization methods. VPMBench introduces a standardized interface for prioritization methods and provides a plugin system that makes it easy to evaluate new methods. It supports different input data formats and custom output data preparation. VPMBench exploits declaratively specified information about the methods, e.g., the variants supported by the methods. Plugins may also be provided in a technology-agnostic manner via containerization.

**Conclusions:**

VPMBench significantly simplifies the evaluation of both custom and published variant prioritization methods. As we expect variant prioritization methods to become ever more critical with the advent of whole-genome sequencing in clinical diagnostics, such tool support is crucial to facilitate methodological research.

## Background

Recent improvements in price and scalability of next-generation sequencing (NGS) make whole-exome and whole-genome sequencing (WES, WGS) feasible in clinical practice (e.g. [1, 2]). However, diagnosing WES and WGS cases is challenging. In a typical WES case, 25,000–75,000 of a patient’s genetic variants have to be narrowed down to a handful of relevant variants (e.g. [3, 4]; WGS increases these numbers ca. 30-fold). Even after considering population allele frequency [5], gene-phenotype associations [6], or other variant annotations, dozens of potentially relevant variants may be found in a case. A clinician has to manually review these variants in a time-consuming and cumbersome process [7]. Variant prioritization methods are being developed to allow the clinican to focus on those variants that are likely disease-causing [8].

Variant prioritization methods are calculating numerical scores predicting the functional impact of the patient’s variants [9]. For this, the methods make assumptions about how the degree of pathogenicity of a variant can be recognized [8]. For example, variants in highly conserved DNA regions are likely to be more damaging than variants in less conserved regions, or common variants in a large population are likely to be benign. The pathogenicity of a variant is typically expressed as a *score*. It is calculated by algorithms like ontology propagation [10], decision trees [11], or Hidden Markov models [12], which in turn have been trained or calibrated with data from public databases (e.g., ClinVar [13], HPO [14]). The scores have to be interpreted using cutoffs for pathogenicity specified by the authors of the respective method to classify the variants as *benign* or *pathogenic* [9]. The prioritization methods further differ in terms of their required input format for the variants and their software stack, e.g., fathmm-MKL [15] requires Python version 2.7 and uses tabix [16] whereas CADD [17] relies on bioconda [18] and snakemake [19] to orchestrate a combination of various scripts to retrieve the scores.

Each time a new method is developed, whether based on updated or new databases or by using a different algorithm, its effectiveness in identifying pathogenic variants needs to be evaluated based on different data sources, and, ideally, also compared to already existing methods [12, 17, 20]. Identifying the most suitable method for disease-specific gene panels [21, 22] or specific variation types, e.g., single nucleotide polymorphisms (SNP) or short insertions or deletions (INDELS) [23], also requires an evaluation and comparison of these methods.

A thorough evaluation of variant prioritization methods takes significant effort because

1. the evaluation input data, consisting of the variants and their expected classification, needs to be converted into valid input for each method (e.g., VCF- or CSV-files),
2. the different software stacks of the methods have to be invoked individually, and
3. their outputs have to be converted into a common format, which can serve as the basis for comparison (e.g., by calculating sensitivity and specificity or plot receiver operator characteristic (ROC) curves).

These steps need to be repeated for each performance evaluation study.

To facilitate the evaluation of variant prioritization algorithms, we propose VPMBench. VPMBench  is a test bench that allows us to compare the performance of prioritization methods with little manual intervention by automating the steps outlined above. By this, the user can focus on compiling suitable evaluation datasets, developing a new variant prioritization method, and interpreting the results.

## Implementation

The overall architecture of our test bench assembles a data pipeline [24] in which the variant prioritization methods are integrated as plugins.

To use our pipeline, the user provides the evaluation input data and specifies plugins, performance summaries, and metrics of interest. Our pipeline then parses the evaluation input data, loads and invokes the plugins, collects their output, and calculates the summaries and metrics.

**Fig. 1:**
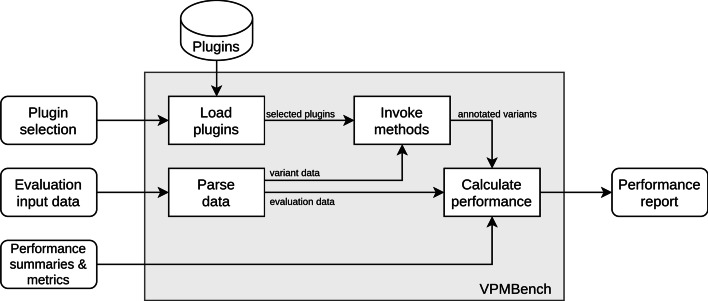
Overview of the VPMBench architecture

The results are passed back as a performance report to the user for further processing, e.g., generating visualizations or analyzing the results. An overview of the pipeline is shown in Fig. 1.

We implemented our VPMBench test bench using Python [25]. Thus, VPMBench test bench can be integrated into existing Python projects using the provided API. The source code can be found at https://github.com/IDEA-PRIO/VPMBench. A thorough documentation is available at https://vpmbench.readthedocs.io/en/latest/.

In the following, we give a short overview of how the different steps of our pipeline and the plugin system have been implemented.

### Input processing

**Table 1:** Format of the evaluation data

Attribute	Name	Description	Values
Unique Identifier	UID	The unique numerical identifier of the variant	${\mathbb {N}}^{\ge 0}$
Reference Genome	RG	The reference genome used to call the variant	String
Chromosome	CHROM	The chromosome in which the variant is found	$\{1,\dots ,22,\text {X},\text {Y},\text {M}\}$
Position	POS	The 1-based position of the variant within the chromosome	${\mathbb {N}}^{\ge 1}$
Reference	REF	The reference bases	$\{\text {A},\text {C},\text {G},\text {T}\}^*$
Alternative	ALT	The alternative bases	$\{\text {A},\text {C},\text {G},\text {T}\}^*$
Variation type	TYPE	The variation type of the variant	$\{\text {SNP},\text {INDEL}\}$
Classification	CLASS	The expected classification of the variant	$\{\text {benign}, \text {pathogeneic}\}$

In the first step, we transform the evaluation input data that contains the variant data and their expected classifications into an internal representation described in Table 1. In the resulting evaluation data, we assign each entry a unique numerical identifier (UID), allowing us to reference the individual entries in our pipeline. Currently, our test bench provides generic extractors for arbitrary VCF- and CSV-based input formats. Using these extractors, we implemented extractors for two publicly available variant-disease databases, i.e., ClinVar [13] and the VariSNP benchmark database suite [26]. Finally, we validate the evaluation data according to the constraints shown in Table 1. If the validation of the evaluation data fails, we report an error to the user requiring to revise the evaluation input data. All variant attributes in the evaluation data except the classification, i.e., UID, RG, CHROM, POS, REF, ALT, are forwarded as variant data to the methods.

### Integrating variant prioritization methods

Variant prioritization methods are integrated as plugins, allowing to include new methods without changing the pipeline code. Each plugin consists of a *manifest* file and integration code for a method’s custom processing logic.

The *manifest* contains a declarative specification of meta-information about the method, i.e., its name, version, supported variation types, cutoff, supported reference genome, and the versions of associated databases and datasets. It also specifies an entry point to the processing logic for our pipeline. The manifest is used to ensure that the evaluation is limited to variation types supported by the method so that VPMBench only calls it with valid data and can thus generate meaningful evaluation reports.

The custom processing logic is invoked as part of our pipeline to calculate a variant’s score. To ensure compatibility of the logic with the rest of our pipeline, we define an interface that accepts variant data as input and returns a tuple (UID,score) for each variant.

The processing logic can be implemented from scratch using Python or be reused from Docker images. When using Python, VPMBench loads and executes the Python file specified as the entry point in the manifest. To reuse a Docker image the user specifies

- the name of the image,
- format and file paths of input and output files, and
- optional bindings to local files separating the processing logic in the image from required databases or indices, e.g, calculated by tabix [16], and
- a command which is executed to invoke the method in the Docker container (see Listing 1).

Our test bench uses this information to write the variant data in the expected input format of the method, to mount the input/output files and bindings, to run the Docker container, and to parse the output file for returning a set of UID and score pairs.

The key difference between the two approaches is the environment in which the method is executed. The Python code is executed in the same environment as the pipeline and thus allows to access methods under local development. In contrast, Docker containers provide a dedicated virtualized environment preventing any conflicts with locally installed software, e.g., conflicting Python versions [27], and also allow implementing the logic in any programming language as long the implementation adheres to the interface described above. Currently, we provide a Docker-based plugin for CADD [17] and Python-based plugins for the non-coding and coding scores from fathmm-MKL [15] and 30 scores from the dbNSFP 4.1a [28] (without fathmm-MKL and CADD).

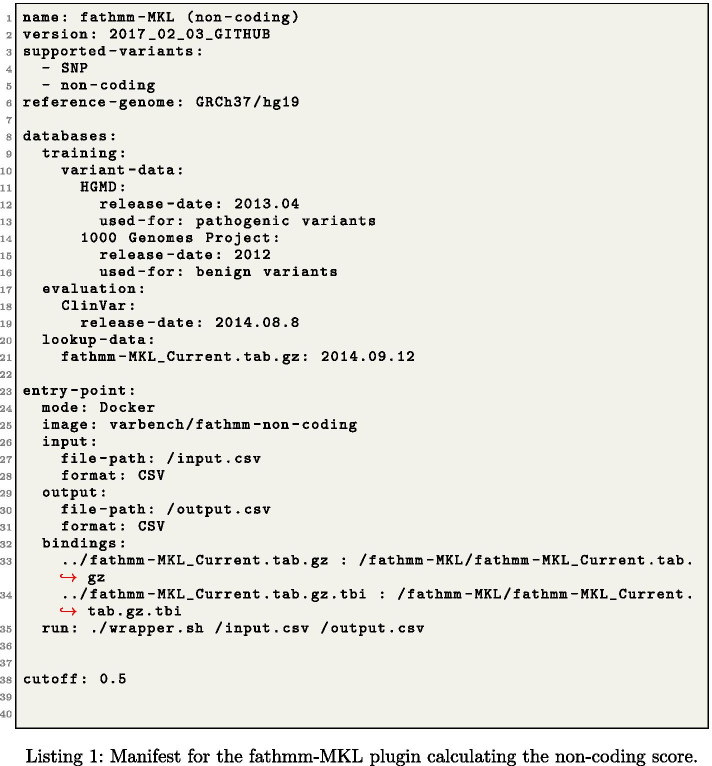



### Invoking the variant prioritization methods

To invoke the variant prioritization methods, we rely on our plugin-based system which automatically discovers available plugins based on the *manifest* files and filters the plugins according to the plugin selection. The plugin selection is specified as a boolean function. This function has to return True when the plugin shall be invoked (see Listing 2).

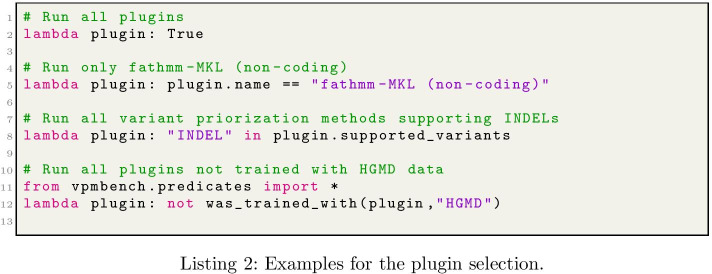



Before running the custom processing logic of the plugins, we check the selected plugins for compatibility with the variant data by comparing the supported variation types and reference genome from the manifest with the corresponding information from the data. If this check fails, we report an error to the user requiring to revise the evaluation input data. For example by liftover the genomic coordinates of the variants to the supported reference genome or remove invalid variation types. Next, we interpret the specified entry points from the plugins and run the custom processing logic of the methods in parallel. The outputs are collected and validated to ensure that each UID got a numerical score assigned, and are then used to annotate the variants with the scores.

### Calculating performance summaries and performance metrics

Variant prioritization methods calculate scores. They are interpreted using the cutoff of the respective method to classify the variants as benign/neutral or pathogenic/deleterious. Thus, we can assess the performance of variant prioritization mechanisms similar to binary classifiers [29–31]. We first calculate performance summaries, e.g., confusion matrices or receiver operator characteristic (ROC) curves [32, 33], by comparing the predicted and expected classification of variants. We calculate performance metrics quantifying the performance based on the performance summaries, e.g., sensitivity (true-positive rate) or specificity (true-negative rate). The performance summaries and metrics of different prioritization methods can then be compared, e.g., to identify the method with the smallest type-2 error (pathogenic variants are incorrectly classified as benign variants) or to identify the optimal cutoffs for the methods.

In our pipeline, the expected classes are given as labels in the evaluation data and thus cannot be compared directly with the numerical scores from the annotated variant data. Therefore, we apply the following label encoding to the expected classes: benign variants are encoded with 0 and pathogenic ones with 1. The calculated scores are interpreted using the cutoffs for pathogenicity. Scores smaller than the cutoff are encoded as 0 and those larger than the cutoff as 1. The cutoff for each method is part of the plugin’s manifest. Also, the user can vary cutoffs to analyze the impact of different cutoffs on the method’s performance, e.g., by ROC curves. The calculated summaries and metrics and the information from the plugin manifest, are passed to the user as performance reports. The plugin information serves as documentation on which basis the summary was calculated.

Currently, we support calculating sensitivity, accuracy, precision, recall, negative predictive value, specificity, concordance, Matthews correlation coefficient, and the area under ROC curves as metrics for the variant prioritization methods. Further, we also support calculating confusion matrices, ROC curves, and precision-recall curves as performance summaries. However, the set of metrics can be extended easily.

## Results

In the following, we demonstrate the functionality of our test bench by comparing the performance of three prioritization methods. The code and data used for the case studies is available under https://doi.org/10.5281/zenodo.5167117.

### Case study 1: comparing CADD, fathmm-MKL (coding), and fathmm-MKL (non-coding)

CADD and fathmm-MKL are widely used for prioritizing variants. Fathmm-MKL supports the calculation of two scores, i.e., for coding and non-coding regions.

Each method is integrated as a Docker-based plugin in our pipeline. We use the first 500 benign and 500 pathogenic SNPs from ClinVar (release date: 2021-02-13) for the GRCh37 reference genome as the evaluation input data. An overview of the Python script using VPMBench, plotting the summaries, and reporting the metrics is shown in Listing 3.

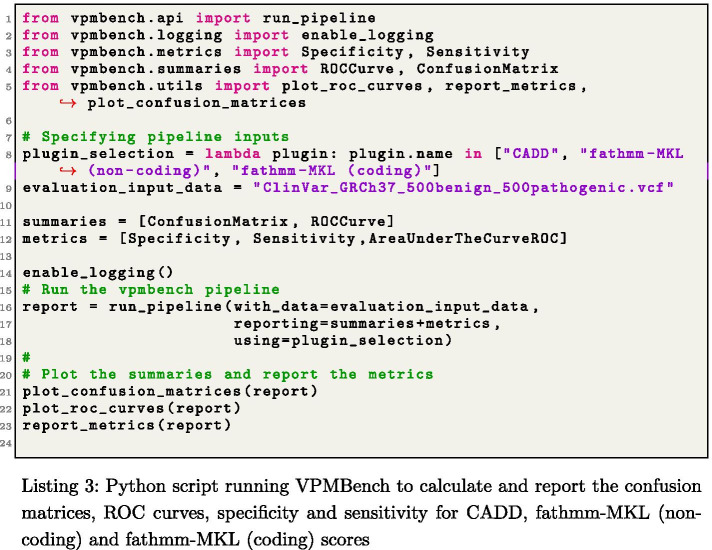



**Fig. 2:**
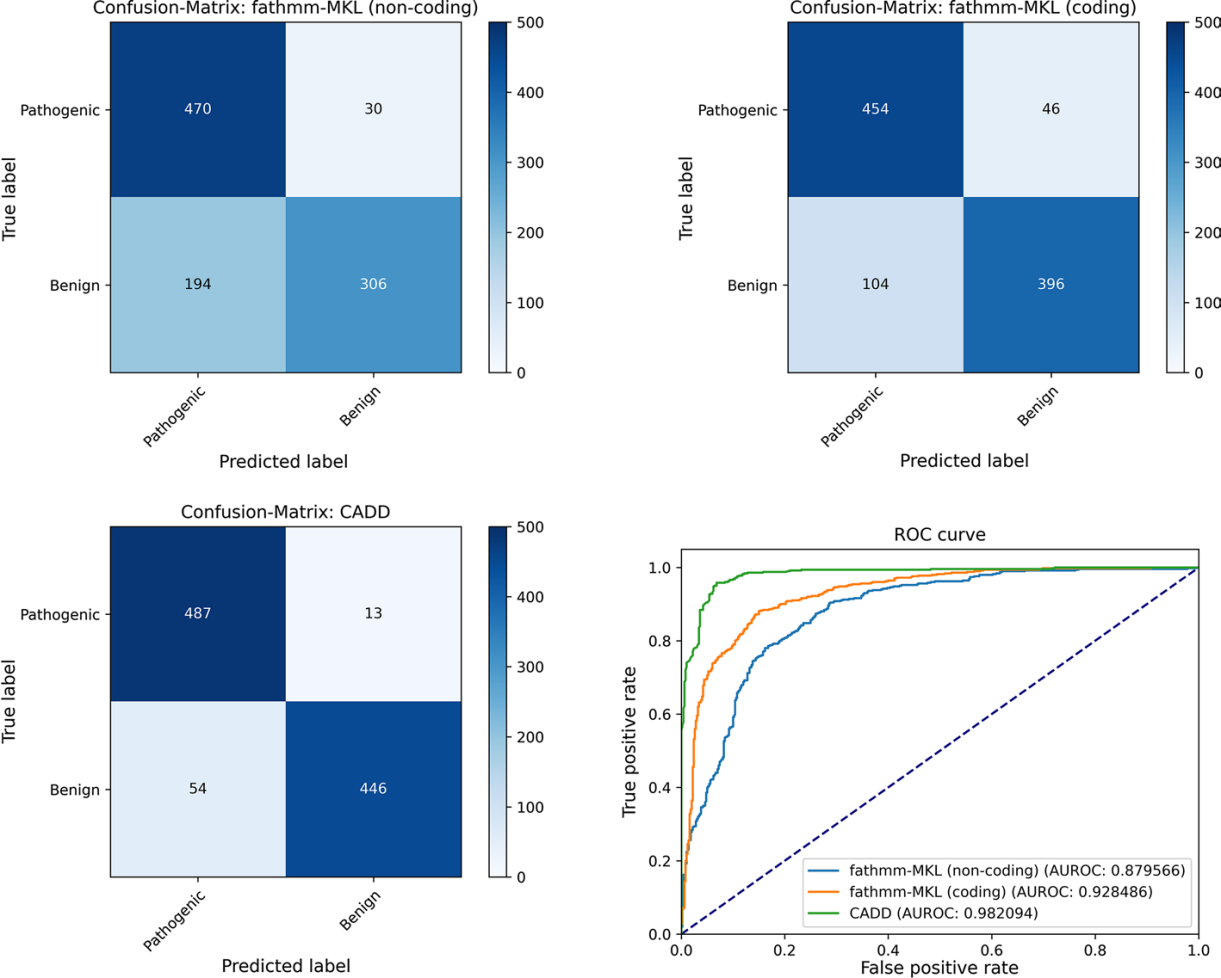
Plots of the confusion matrices and ROC curves for fathmm-MKL (non-coding) and fathmm-MKL (coding), and CADD scores

The Python script starts with specifying the plugin selection as a boolean function returning True so that all plugins are executed. Next, the file path for the evaluation input data is given, followed by the summaries and metrics that should be calculated (Line 8–11). After this, input data are automatically transformed, plugins selected, the variant data are annotated with the scores, and summaries and metrics are calculated (Line 14). Finally, we plot the summaries (Line 19–20) and report the metrics (Line 21). The resulting plots are shown in Fig. 2 and the reported metrics are shown in Table 2.

**Table 2:** The specificity, sensitivity and AUROC reported for the fathmm-MKL (non-coding) and fathmm-MKL (coding), and CADD scores

Method	Sensitivity	Specificity	AUROC
fathmm-MKL (non-coding)	0.612	0.940	0.879566
fathmm-MKL (coding)	0.792	0.908	0.928486
CADD	$\mathbf {0.892}$	$\mathbf {0.974}$	$\mathbf {0.982094}$

Bold indicates best performance

The confusion matrices in Fig. 2 show that the CADD score performs better than the fathmm-MKL scores since 935 of the 1000 variants are correctly classified while also minimizing the type-2 error: only 13 pathogenic variants are incorrectly classified as benign. In contrast, only 850 variants would be correctly classified based on the fathmm-MKL coding score and 776 variants based on the fathmm-MKL non-codings score. Also, the coding score has the highest type-2 error as 46 pathogenic variants would be classified as benign. Another approach to summarize the performance is to use ROC curves, where the false-positive rate (1-specificity) and true-positive rate (sensitivity) of the methods are plotted against each other for various cutoffs [32, 33]. In the resulting plot, the curves of the methods with a high discriminate capacity are closer to the top left of the graph, as a good classifier achieves a high true-positive rate at a low false-positive rate. The ROC curves in Fig. 2 show that CADD has the highest discriminate capacity among the prioritization methods. The same conclusion can be drawn by comparing the area under the ROC curves (AUROC) of the different prioritization methods (see Table 2). By comparing sensitivity and specificity of the methods as given in Table 2, measuring how effectively pathogenic and benign variants can be classified, we see that CADD performs better than both fathmm-MKL methods on our evaluation data. Further, we see that the specificity of the fathmm-MKL non-coding score is higher than for the coding score of the same method, but the sensitivity is lower for the non-coding score than for the coding score. A subsequent analysis using additional information about the variants might allow correlating these observations with specific attributes of the misclassified variants, e.g., as done in [34] for types of amino acid substitution.

### Case study 2: comparing the concordance of CADD, fathmm-MKL (coding), and fathmm-MKL (non-coding) for annual ClinVar releases

When looking at the publication dates of these methods, we see that the current version of CADD was trained in 2020 and fathmm-MKL in 2014. From this, one might ask how the performance of these methods change over time.

**Fig. 3:**
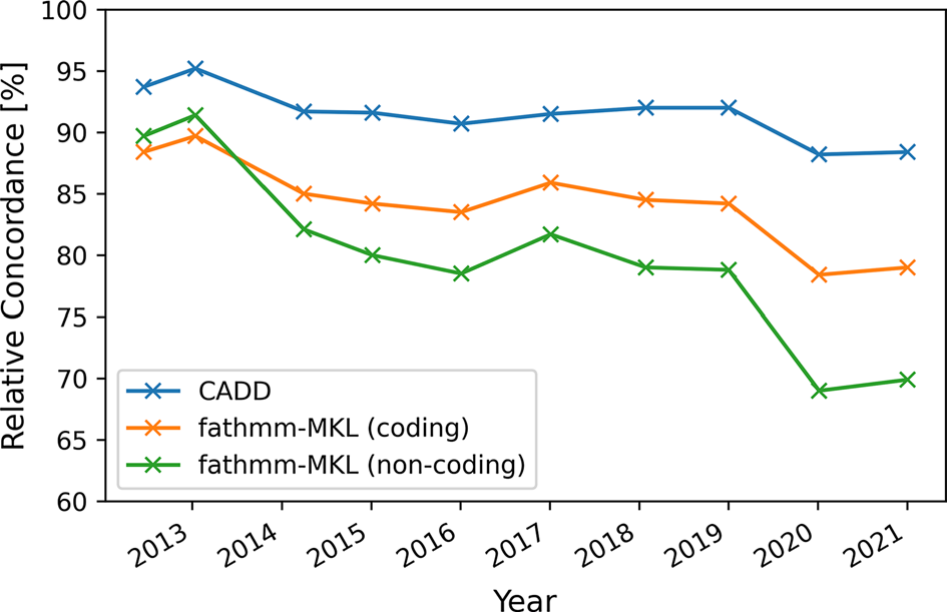
Relative concordance of CADD, fathmm-MKL (coding), and fathmm-MKL (non-coding) for annual ClinVar releases

To illustrate how VPMBench  can answer this question, we first downloaded the GRCh37 ClinVar releases for 2012 to 2021 and filtered for SNPs classified as benign or pathogenic. Second, VPMBench  was used to measure the concordance, i.e., the number of variants correctly classified as benign or pathogenic, for each method and filtered ClinVar release. Next, we calculated the relative concordance, i.e., the percentage of correctly classified variants out of the total number of variants in the respective ClinVar release. Finally, we aggregated the results (see Table 3). A graphical overview of the results is shown in Fig. 3.

**Table 3:** The number of variants and measured concordance of the fathmm-MKL (non-coding), fathmm-MKL (coding), and CADD scores for the different ClinVar releases

Year	#Variants	fathmm-MKL (non-coding)	fathmm-MKL (coding)	CADD
2012	10,295	9,235 (89.7%)	9,099 (88.4%)	9,651 (93.7%)
2013	10,484	9,579 (91.4%)	9,402 (89.7%)	9,978 (95.2%)
2014	14,960	12,288 (82.1%)	12,723 (84.2%)	13,716 (91.7%)
2015	18,725	14,981 (80.0%)	15,762 (84.2%)	17,159 (91.6%)
2016	24,449	19,188 (78.5%)	20,416 (83.5%)	22,183 (90.7%)
2017	31,726	25,910 (81.7%)	27,263 (85.9%)	29,044 (91.5%)
2018	56,115	44,351 (79.0%)	47,414 (84.5%)	51,650 (92.0%)
2019	63,354	49,907 (78.8%)	53,362 (84.2%)	58,297 (92.0%)
2020	111,289	76,754 (69.0%)	87,302 (78.4%)	98,160 (88.2%)
2021	134,384	93,879 (69.9%)	106,179 (79.0%)	118,734 (88.4%)

In the results, we can make the following observations: (1) the relative concordance decreases overtime for all methods, and (2) CADD always has a higher relative concordance than the fathmm-MKL methods.

 To explain these observations, we must consider that the number of SNPs classified as benign or pathogenic in ClinVar has increased thirteenfold from 2012 to 2021. Each ClinVar release provides more data than the previous one and, thus, the measured performance more and more approximates the “actual” performance of the prioritization methods determined by the annotations, i.e., features and the machine-learning models used during their development process. The features and the machine-learning models used are also explaining the performance difference of these methods. CADD uses a logic-regression model, whereas fathmm-MKL uses a support vector machine model. Thus, the performance difference can be partially explained due to the capability of these methods to classify the variants based on the annotations used in the training procedure. When comparing the number of different features used to train the machine-learning models, we find that CADD was trained on $\approx 100$ features with $\approx 17\times 10^6$ variants in 2020. In contrast, fathmm-MKL methods were trained on $\approx 750$ features with $\approx$ 8000–21,000 variants in 2013. The performance of machine-learning models is highly affected by the so-called *curse of dimensionality* according to which a large amount of training data is necessary to train a machine-learning model on large feature spaces [35]. Thus, we suspect that insufficient training data was used to train the fathmm-MKL models to achieve comparable performance with CADD. Finally, we notice that an unbiased evaluation of these methods would also require removing the variants used to train the machine-learning models. Unfortunately, the original training data of CADD and fathmm-MKL is not available.

In general, these results show that it makes sense to check state-of-the-art prioritization mechanisms against the latest clinical data regularly. VPMBench  can automate this procedure.

## Discussion

Next, we describe how VPMBench  can support input formats other than VCF and CSV and discuss related approaches.

### Supporting other evaluation data input formats

In our pipeline, we convert the variant information into the expected input formats for prioritization methods, invoke them and compare their results with the expected classifications of the variants to calculate performance summaries and performance metrics. Thus, we rely on evaluation data input formats, e.g., VCF or CSV, that are able to store the variant information (CHROM, POS, $\dots$) along with their expected classifications (CLASS).

Formats such as SPDI [36], VRS [37] or HGVS [38] are designed to only represent the variant information.

To generate the required input files for our pipeline, the user can use existing tools, such as bcftools [39] or VCFtools [40], to convert the variant information into a CSV- or VCF file. The resulting variants in these files then have to be annotated with their expected classification using information from variant-disease databases such as ClinVar. This step can also be supported by using annotation pipelines such as ANNOVAR [41].

### Related work

Similar procedures, where presumably several scripts are combined to transform the input, to invoke different methods, and to calculate the performance summaries and metrics, have been repeated many times, e.g., for evaluating a new variant prioritization method [12, 17, 20] or investigating the performance of methods for a specific type of variant [21, 22, 34] or disease [42, 43]. In contrast to these “ad-hoc implementations”, our test bench provides a structured pipeline automating these steps while allowing to integrate methods via plugins.

For annotating the variants with scores, alternatives to our plugin-based approach exist in the form of pre-computed databases, dedicated web services provided by authors to test their methods, and variant annotation pipelines.

Databases, e.g, dbNSFP [28], focus typically on a specific type of variation, e.g., non-synonymous single-nucleotide variants, they are not applicable for other types of variations [41], e.g, INDELs. Thus they are not suitable for a performance analysis covering a range of variation types.

The use of web services [44–46] introduces additional load and reliability problems, as it relies on an externally managed service while implicitly assuming that services are available when needed, that they do not change communication interfaces or versions, and that they can handle the required amount of evaluation input data.

One could also use variant annotation pipelines, such as ANNOVAR [41] or the Ensembl Variant Effect Predictor [47], allowing to annotate variants with a variety of information including, gene-based annotations, region-based annotation, and prioritization scores. These approaches share architectural similarities with our test bench as they support annotating variant data from different input formats and including new annotations sources as pre-computed databases [41] or plugins [47]. However, they aim at providing a one-stop source for annotating variants as part of a sequencing pipeline and at supporting a biologist in interpreting the variants [47] or analyzing population samples [48]. In contrast, our pipeline focuses on facilitating and automating the evaluation of prioritization methods.

## Conclusions

Here, we presented VPMBench, a test bench automating the evaluation of variant prioritization methods. The test bench is implemented as a data pipeline with a plugin system. *Manifest* files, in which meta information and the entry points are specified, allow a flexible integration of additional prioritization methods. The *Manifest* files are interpreted by VPMBench to convert the evaluation input data into the expected format for the methods, to invoke the methods, to read the methods’ output, and to interpret the scores for calculating performance summaries and metrics for each method. Despite focusing on the evaluation of prioritization methods trying to classify variants as benign/pathogenic, VPMBench also provides prototypical support for non-binary methods classifying according to the ACMG guidelines [49, 50] or the ClinVar database [13] (see Documentation). By this, VPMBench supports performance studies comparing the performance of various methods as well as the development of new prioritization methods.

For future development, we plan to include further state-of-the-art variant prioritization methods into our test bench for comparison. In particular, we aim at ensemble-based methods [51], which combine multiple scores in order to produce superior classificators [52, 53]. Additionally, we aim to improve our testbench by extending the extractor interface so that meta-information about the variants, e.g., whether a variant is coding/non-coding, can be integrated as variant attributes as part of the evaluation data. This information will then be used to implement additional features, e.g., a warning mechanism that warns the user when non-coding prioritization methods tries are used to calculate scores for coding variants. Moreover, we want to include an automatic liftover for variants. Recent studies [54, 55] suggest that liftover can produce accurate results. Therefore, we want to investigate how an automatic liftover, as it is also done in variant annotation pipelines, affects the performance of variant prioritization methods.

## Availability and requirements

Project name: VPMBench

Project home page: https://github.com/IDEA-PRIO/VPMBench

Operating system(s): Linux

Programming language: Python

Other requirements: Docker, Python libraries (Pandas, Pandera, Scikit-learn, Docker-SDK, Matplotlib, PyYAML, Numpy, PyVCF)

License: MIT

Any restrictions to use by non-academics: none

## Data Availability

The Source code is available at GitHub https://github.com/IDEA-PRIO/VPMBench. The documentation is available at https://vpmbench.readthedocs.io/en/latest/. The datasets and code used in the case studies is available at https://doi.org/10.5281/zenodo.5167117.

## Abbreviations

NGS: Next-generation sequencing
WGS: Whole-genome sequencing
WES: Whole-exome sequencing
SNP: Single nucleotide polymorphism
INDELS: Insertions or deletions
ROC: Receiver operator characteristic
